# Effect of Functional Rehabilitation on Performance of the Star Excursion Balance Test Among Recreational Athletes With Chronic Ankle Instability: A Systematic Review

**DOI:** 10.1016/j.arrct.2021.100133

**Published:** 2021-05-21

**Authors:** Leanne Ahern, Orla Nicholson, Declan O'Sullivan, Joseph G. McVeigh

**Affiliations:** Discipline of Physiotherapy, School of Clinical Therapies, College of Medicine and Health, University College Cork, Cork, Ireland

**Keywords:** Ankle, Postural Balance, Rehabilitation, Systematic Review, MDC, minimal detectable change, SEBT, Star Excursion Balance Test, WBVT, whole-body vibration training

## Abstract

**Objective:**

To determine (1) the effectiveness of rehabilitation for chronic ankle instability as measured by the Star Excursion Balance Test (SEBT) and (2) the relative efficacy and the long-term effects of these rehabilitation interventions.

**Data Sources:**

Ten electronic databases were searched (2009-2019).

**Study Selection:**

Included articles were randomized controlled trials in English investigating recreational athletes aged ≥18 years with chronic ankle instability. At least 1 functional rehabilitation intervention had to be included and the SEBT test (or the modified version) used as an outcome measure.

**Data Extraction:**

Two researchers (L.A., O.N.) extracted data regarding participant demographics; intervention characteristics; trial size; and results at baseline, postintervention, and at follow-up, where appropriate.

**Data Synthesis:**

A systematic review and narrative synthesis was conducted. Methodological quality of included studies was assessed using the Cochrane Risk of Bias Tool and the van Tulder scale. The review was registered with PROSPERO (ID: 164468). Ten studies (n=368), 2 high-quality, 1 moderate-quality, and 7 low-quality, were included in the review. Interventions included balance training, strength training, vibration training, and mixed training. Results suggest that rehabilitation of chronic ankle instability that includes wobble board exercises (average percentage change: 14.3%) and hip strengthening exercises (average percentage change: 12.8%) are most effective. Few studies compared different types of rehabilitation for chronic ankle instability. However, improvements on the SEBT suggest that a rehabilitation program focusing on wobble board training and hip strengthening performed 3 times weekly for 4-6 weeks is the optimal rehabilitation program to improve dynamic postural control in recreational athletes with chronic ankle instability.

**Conclusions:**

Few studies directly compared different rehabilitation interventions, and there was limited long-term follow-up; therefore, the relative efficacy of different rehabilitation programs remains unclear. However, it seems that rehabilitation of chronic ankle instability should include proprioceptive and strengthening exercises of relatively short duration.

A lateral ankle sprain involves the ankle rolling inward at a high velocity, damaging the lateral ligament complex.^1^, ^2^, ^3^, ^4^ A total of 85% of lateral ankle sprains result from excessive inversion.^2^^,^^5^ Lateral ankle sprains account for approximately 30% of all injuries^6^, ^7^, ^8^ and frequently occur among sporting individuals.^9^, ^10^, ^11^ Lateral ankle sprains damage the mechanoreceptors in the tissues surrounding the ankle and^12^, ^13^, ^14^, ^15^ potentially lead to residual pain, “giving away,” and functional impairments.^16^ Risk of reinjury is estimated up to 73%,^17^ with approximately 31%-40% developing chronic ankle instability.^16^^,^^17^ Lateral ankle sprains are linked to high economic costs and reduced work productivity,^18^ emphasizing the economic burden of this injury.

Chronic ankle instability commonly consists of reoccurring ankle sprain, residual symptoms, and episodes of “giving way”^19^ and encompasses functional and mechanical ankle instability.^19^ Functional instability incorporates muscle strength deficiencies and an impaired proprioceptive system,^12^^,^^20^ resulting in altered sensorimotor and postural control.^21^ Ankle sensorimotor control incorporates muscle activity, which influences ankle stability.^22^ Deficits in peronei muscle function are common after a sprain.^23^ These muscles prevent potential injury by resisting the inverting forces that may cause excessive inversion.^24^ Impaired dynamic postural control results from diminished strength, range of motion, neuromuscular control, and proprioception.^17^

The Star Excursion Balance Test (SEBT) is a widely used, reliable, and valid measure of dynamic postural control.^25^^,^^26^ It is an inexpensive, simple measure^27^ and has been extensively investigated among individuals with chronic ankle instability.^17^^,^^28^, ^29^, ^30^, ^31^, ^32^, ^33^, ^34^ A modified version, known as the Y Balance Test is also commonly used.^35^, ^36^, ^37^, ^38^, ^39^, ^40^

Rehabilitation programs including balance,^35^, ^36^, ^37^^,^^41^, ^42^, ^43^, ^44^ strength,^37^, ^38^, ^39^ vibration,^45^ and mixed training^40^^,^^46^^,^^47^ have been investigated. However, evidence is conflicting regarding which intervention type is the most efficacious.

Balance training improves an individual's ability to maintain center of gravity and posture by challenging the vestibular and musculoskeletal systems.^48^ It has been reported that balance training can improve dynamic postural control among individuals with chronic ankle instability.^43^^,^^49^^,^^50^ McKeon et al^43^ conducted a high-quality study investigating the effects of a 4-week balance program among adults with chronic ankle instability and reported significant improvements in the intervention group for self-reported disability and postural control.^43^

Strength training involves exerting force in an attempt to surmount resistance, leading to greater recruitment and stronger synchronization of muscle fibers,^48^ which improves neuromuscular control and muscular development.^51^, ^52^, ^53^, ^54^, ^55^, ^56^ Smith et al^57^ conducted a high-quality study investigating the effects of a 6-week strength program among individuals with chronic ankle instability. They reported significant improvements in evertor strength in the intervention group and concluded that an effective strength program should be challenging and multiplanar to improve strength and prepare the ankle for return to regular activity.^57^

Whole-body vibration training (WBVT) involves mechanical oscillations transmitted from a vibration platform that alters joint mechanoreceptors, muscle spindles, power, and strength performances,^58^ but there is limited research exploring WBVT for chronic ankle instability, Ray^59^ conducted a moderate quality meta-analysis (n=4) comparing the effectiveness of WBVT to wobble board rehabilitation. These authors concluded that wobble board training was more effective for improving dynamic postural control in recreational athletes with chronic ankle instability.

In 2010, Webster and Gribble^60^ systematically reviewed functional rehabilitation literature for chronic ankle instability (n=6 randomized controlled trials). Their findings suggested that all functional rehabilitation interventions significantly improved postural control.^60^ However, they did not compare interventions for their relative efficacy, nor did they assess follow-up periods to determine the long-term effects. This current review provides an up-to-date review of the most recent literature (the last 10 years) exploring the optimal rehabilitation parameters, as measured by the SEBT, to assist clinicians with the conservative management of chronic ankle instability.

The aim of this review is to determine the effectiveness of functional rehabilitation for improving dynamic postural control, as measured by the SEBT, among recreational athletes with chronic ankle instability, with specific consideration for the relative efficacy and long-term effects of interventions*.*

## Methods

This review was conducted according to the Preferred Reporting Items for Systematic Reviews and Meta-Analyses guidelines.^61^ The review protocol was registered with PROSPERO (ID: 164468, awaiting confirmation of registration).

### Data sources and searches

Two researchers (L.A., O.N.) conducted an electronic search of 10 databases from 2009-2019 to update the literature after the last systematic review^60^ conducted on the topic.

Box 1 displays the databases searched and the keywords used. Reference lists of relevant articles were checked to identify further eligible studies. Titles and abstracts of potential eligible studies were screened (L.A., O.N.). Any disagreements were resolved by an additional researcher (D.O. or J.M.).

Box 1 **Search Strategy**

**Table:** 

Databases: - EBSCO - MEDLINE - SPORTDiscus - CINAHL - Web of Science - PubMed - Embase - Scopus - Google Scholar - Cochrane Library
Search keywords:
1. [reach OR performance]
2. [“chronic ankle instability” OR cai OR “functional instability” OR “non-copers”]
3. [“star excursion balance test” OR sebt OR “Y balance test”]
4. [functional rehabilitation” OR intervention OR exercise OR “closed-chain exercise”]
5. 1 AND 2 AND 3 AND 4
6. [“randomized controlled trial”]
7 5 AND 6

Alt-text: Unlabelled box

### Study selection

Full-text randomized control trials published in English were included. For the purposes of this review recreational athletes were self-reported or defined as completing at least 20 minutes of moderate to high intensity physical activity 3 times per week.^62^ It is recognized, however, that the definition of recreational athletes varies across studies. Brown et al,^62^ for example, defined recreational athletes as those who participate in at least 20 minutes of physical activity 2 times per week. However, Ray^59^ defined recreational athletes as individuals participating in more than 1.5 hours of moderate to vigorous physical activity per week. A previous study by Sierra-Guzman et al^47^ did not even define recreational athletes. Functional rehabilitation was defined as “dynamic, closed-kinetic-chain activity other than quiet standing.”^37^^(p99)^ Included trials were required to report the SEBT or Y Balance Test reach performances. Both short-term (6-12wk) and long-term (12+wk) follow-up studies were included.

Table 1 displays the eligibility criteria for the included studies. The primary outcome of interest was improvements in the SEBT performances, expressed as a percentage of change relative to preintervention.

**Table 1:** Eligibility criteria

Inclusion Criteria	Exclusion Criteria
Study design: randomized control trials Population of interest: Recreational athletes, any sex, aged 18+ y with CAI Intervention: At least 1 form of functional rehabilitation (eg, balance, strength, vibration, mixed training) Comparison or control group: Control group was required to fulfill at least 1 of the following conditions: a) Recreational athletes without CAI b) An active comparator, usual care, or a sham group c) If the entire sample consisted of recreational athletes with unilateral CAI, the contralateral uninjured limb was the control, or d) If the entire sample involved recreational athletes with bilateral CAI, the control limb was specified.	Trials were excluded if the recruited participants involved any of the following conditions: - Aged <18 y - Not recreational athletes - Injury <4 wk ago - Multiple injuries - Nonfunctional CAI - Neurologic impairments - Vestibular impairments - Upper respiratory infection - Ear infection - Other conditions that affect balance - Previous stabilization procedure - Previous fixation surgery. Other reasons for exclusion included the following: - Control criteria not met - Postintervention results not reported - Full-text article not available

Abbreviation: CAI, chronic ankle instability.

### Data extraction

Two researchers (L.A., O.N.) extracted data regarding participant demographics, intervention characteristics, trial size, baseline and postintervention results, and follow-up results where relevant.

### Risk of bias assessment

Two independent reviewers (L.A., O.N.) assessed each study using the Cochrane Risk of Bias Tool^63^ and the van Tulder scale.^64^^,^^65^ As recommended in the Cochrane Handbook for Systematic Reviews of Interventions^65^ the Cochrane Risk of Bias Tool was used to assess 5 issues associated with risk of bias: sequence generation, allocation concealment, blinding of personnel and outcome assessors, incomplete outcome data, selective reporting, and additional possible threats to validity not previously identified. The van Tulder scale was also included because it assesses both compliance and timing of outcome assessments. Any ambiguity was discussed and a consensus reached, and disagreements were resolved by further discussion with D.O. or J.M.

### Quality assessment

#### Data synthesis

The data synthesis was conducted following the recommended standards of performance outlined by Eden et al^66^: description of the methodological characteristics of selected trials; strengths and limitations of each trial; how the limitations may have influenced the results; the relationship between the study characteristics and reported findings; and the relevance of each trial to its population, control, and outcomes of interest (table 2). Because there are numerous interventions used in the management of chronic ankle instability, with substantial clinical heterogeneity between studies, a meta-analysis was not conducted.

**Table 2:** Quality Assessment Guidelines^66^

Criteria used to determine the quality of the evidence^66^ 1. Adequate randomization 2. Adequate allocation concealment 3. Blinding of assessors 4. Intent-to-treat-analysis 5. Measurement of compliance	
Classification	
High quality	Met 4 of the above 5 criteria (including allocation concealment) and scored at least 5/11 on the van Tulder scale.
Moderate quality	Met 3 of the 5 criteria and scored at least 5/11 on the van Tulder scale.
Low quality	Met ≤2 of the 5 criteria and scored <5/11 on the van Tulder scale.

### Quantifying the magnitude of results

The minimal detectable change (MDC) values outlined by Munro et al^67^ (table 3) were chosen because they include the complete SEBT rather than a subsection and are more conservative. The average percentage change was calculated from the reach distances reported in the studies and compared with the average MDC value for those reach directions.

**Table 3:** Minimal detectable change values^67^

Direction	%
Anterior	6.87
Anteromedial	6.13
Anterolateral	7.71
Medial	7.40
Lateral	7.68
Posterior	7.73
Posteromedial	3.36
Posterolateral	4.28
Composite	7.7*
Complete SEBT average	6.4†
SEBT (A, AM, MED, PM, PL) average	5.61†
Y-balance (A, PM, PL) average	4.48†
Y-balance (AM, MED, PM) average	5.63†

Abbreviations: A, anterior; AM, anteromedial; MED, medial; PL, posterolateral; PM, posteromedial.

⁎ MDC values developed by Hall et al.^38^

† Manually calculated average MDC values for specific directions based on MDC values outlined by Munro et al.^67^

## Results

### Study selection

**Fig 1:**
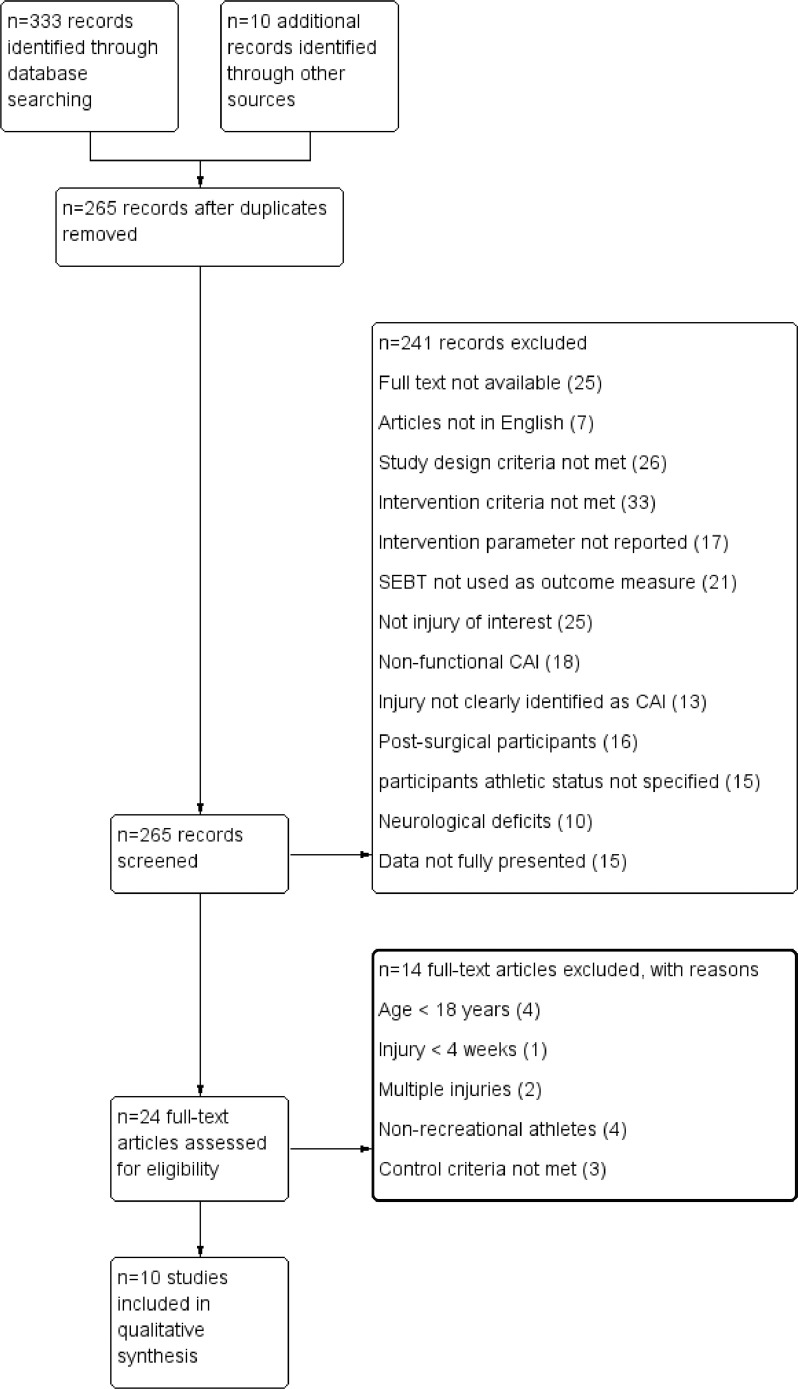
PRISMA flowchart. Abbreviations: CAI, chronic ankle instability; PRISMA, Preferred Reporting Items for Systematic Reviews and Meta-Analyses.

Figure 1 displays the search results and explanations for exclusion. After the database and hand searches, 343 articles were identified; 24 full-text articles were assessed. Ten articles (2010-2018) featuring 368 participants were suitable for this review. Table 4 displays the study characteristics of the included articles*.*

**Table 4:** Characteristics of included studies

Article	Sample			Trial					Follow-up	Conclusions	
Author(s)	Sample Size	Sex	Age (y), mean ± SD	Intervention Type	Treatment Limb	Dosage	Comparison/Control Group	Outcome Measure	Follow-up/Duration	Results/Comments	Limitations
Anguish and Sandrey^35^	18	M:16 F:2	18.38±1.81	PHSB: Exercises included 1) SL hops to stabilization in 4 different directions, 2) Hop to stabilization and reach, 3) Unanticipated hop to stabilization, and 4) SLS activities with eyes open and closed and on compromising surfaces.	Affected limb If a participant reported bilateral ankle instability, the self-reported worse limb was used for training and analysis	30-min supervised sessions, 3 times/wk for 4 wk	SLB: 1) SLS for 60 s, 2) SLS with a ball toss, 3) SLS kicking against resistance in 4 directions, and 4) Step-downs with the single limb in 4 directions.	Y Balance Test	NA	Within-group differences in both groups showed a significant improvement for 3 SEBT reach directions (*P*<.001). Reach distances increased after both balance training interventions with large (PL direction) and moderate to large (A and PM directions) ESs. Between-group ESs were small for all directions, and all 95% CIs crossed 0. A: ES=0.20; 95% CI, −0.72 to 1.13 (favors PHSB group). PM: ES=−0.20; 95% CI, −1.13 to 0.73 (favors SLB group) PL: ES=−0.18; 95% CI, −1.11 to 0.75 (favors SLB group)	1. Results cannot be generalized to other participants with CAI because of small sample size. 2. Lack of participant blinding. 3. No follow-up performed; therefore, the extent to which improvements might lead to a reduction in ankle sprains is unknown.
Burcal et al^36^	24	M:7 F:17	21.3±2.0	Balance sessions included 1) Hop to stabilization in 4 directions, 2) Hop to stabilization and reach in 4 directions, 3) Unanticipated hop to stabilization, 4) Progressive SLS activities, and 5) Progressive SLS activities with eyes closed.	Affected limb If an individual had bilateral instability, the limb with lower FAAM and FAAM-S scores was used as the treatment limb.	20-min supervised sessions performed 3 times/wk for 4 wk	5-min set of STARS treatments including calf stretching, plantar massage, ankle joint mobilizations, and ankle joint traction performed before balance session.	Y Balance Test	1 wk	Between-group ESs ranged from –0.41 for the A direction (favoring the Balance Training group) to 0.25 for PL direction (favoring Balance Training STARS group) immediately posttest; all between- group ESs had 90% CIs that crossed 0. Immediate posttest: Balance Training group A: ES=0.79; 90% CI, 0.10 to 1.49 PM: ES=0.89; 90% CI, 0.19 to 1.60 PL: ES=0.85; 90% CI, 0.15 to 1.55 Balance Training STARS group A: ES=0.54; 90% CI, −0.14 to 1.23 PM: ES=1.43; 90% CI, 0.68 to 2.18 PL: ES=1.35; 90% CI, 0.60 to 2.09 1-wk follow-up: Balance Training group A: ES=0.49; 90% CI, −0.20 to 1.17 PM: ES=0.92; 90% CI, 0.21 to 1.62 PL: ES=1.47; 90% CI, 0.71 to 2.22 Balance Training STARS group A: ES=0.25; 90% CI, −0.43 to 0.92 PM: ES=0.42; 90% CI, −0.26 to 1.10 PL: ES=1.15; 90% CI, 0.43 to 1.88.	1. Small sample size. 2. Lack of participant blinding. 3. Time points of follow-up assessment need to be expanded for future research.
Cloak et al^45^	38	F:38	19±1.1	WBVT with single-leg exercises on a vibration platform (SL heel raises, SL squats).	Affected limb	1 session/wk with increasing duration and frequency over 6 wk. Wk 1 & 2: 30 Hz for 10 min Wk 3 & 4: 35 Hz for 12 min Wk 5 & 6: 40 Hz for 14 min	Normal training regimen; refrained from ankle specific strength/balance training for 6 wk.	SEBT	NA	Significant improvements in A (*P*=.036), AM (*P*=.038), MED (*P*=.047), and AL (*P*=.015) directions in WBVT group compared with control group. No significant between-group differences in PM (*P*=.23), P (*P*=.58), PL (*P*=.23), and L (*P*=.19) directions.	1. No follow-up performed. 2. Sample only included dancers. 3. All female sample.
Cruz-Diaz et al^40^	70	M:35 F:35	30.68±9.37	General strength and coordination training for lower body with additional balance training including 1) Exercise mats (SLS on different surface), 2) Dynair (DLS or SLS, with added throwing exercises), 3) BOSU (same as Dynair), 4) Mini trampoline (same as Dynair; added jump landings), 5) Foam roller (DLS or SLS started with half foam roller and progressed to classic foam roller; added throwing exercises), 6) Resistance bands (resisted DF, PF, INV, and EVN), and 7) Ankle disc (same as Dynair).	Affected limb	3 sessions/wk for 6 wk with progression in intensity every 2 wk. 45 s work to 30 s rest; circuit was completed twice.	General activity combined with strength training for general lower body; instructed to avoid balance training tasks.	Y Balance Test	NA	Within-group differences in experimental group showed a significant improvement for the 3 reach directions (*P*<.001). ESs were moderate in A direction and larger in PM and PL directions in experimental groups, and 95% CIs did not cross 0 for any direction. A: ES=0.66; 95% CI, −4.26 to −3.06 PM: ES=1.38; 95% CI, −4.96 to −3.70 PL: ES=1.83, 95% CI, −5.12 to −3.84. Between-group differences in change scores were significant for all distances (*P*<.001).	1. No follow-up performed.
Hall et al^38^	39	M:17 F:22	18.9±1.3	Strength: resistance band exercises for DF, PF, INV, and EVN. PNF: concentric contraction of the antagonist followed by a concentric contraction if the agonist. D1: dorsiflexion- inversion and plantarflexion-eversion D2: dorsiflexion-eversion and plantarflexion-inversion.	Affected limb If an individual had bilateral instability, the ankle with the highest score on the functional ankle instability questionnaire was considered the treatment limb.	3 sessions/wk for 6 wk	Regular activities avoiding strength or rehabilitative ankle exercises for 6 wk.	Y Balance Test	NA	Composite Y Balance Test did not demonstrate a significant time-by-group interaction (*P*=.08). Neither RBP nor PNF group improved from pretest to posttest. ESs were moderate in both groups, and all 95% CIs crossed 0. RBP: ES=0.6; 95% CI, −0.2 to 1.4 PNF: ES=0.6; 95% CI, −0.2 to 1.4.	1. No follow-up performed. 2. Results displayed as composite score, giving little insight regarding specific direction. 3. Order of reaching directions was not randomized, which may have influences participants performance- learning effect.
Hall et al^37^	39	M:21 F:18	BTP: 23.5±6.5 STP: 24.6±7.7 Control 24.8±9.0	BTP: Exercises included 1) Hop to stabilization, 2) Hop to stabilization and reach, 3) Hop-to-stabilization box drill, 4) Progressive SLS activities with eyes open, and 5) Progressive SLS activities with eyes closed. STP: Based on the RBP and PNF strength protocol used by Hall et al^38^ with the addition of single-leg heel raises on a step.	Affected limb If an individual had bilateral instability, the ankle with the highest score on the functional ankle instability questionnaire was considered the treatment limb	20-min session, 3 times/wk for 6 wk	Mild to moderately strenuous bicycle workout.	Y Balance Test	NA	Within-group differences were noted for both experimental groups (BTP: *P*=.001; STP: *P*=.001). ESs were large for BTP (Hedges’ *g*=0.7) and STP (Hedges’ *g*=0.6) groups.	1. Researchers were not blinded to group allocation.
Linens et al^44^	34	M:6 F:28	Exp: 22.94±2.77 Control 23.18±3.64	Wobble board rehabilitation: 5 levels were available for training with the height of each increased by half inches, heights ranges between 1 and 3 inches.	Affected limb If an individual had bilateral instability, the more symptomatic ankle was chosen.	3 times/wk for 4 wk Five 40-s trials were completed with 1 min of rest in between trials.	No intervention.	SEBT	NA	Within-group differences in experimental group showed a significant improvement for the 3 reach directions (AM: *P*=.016; MED: *P*=.001; PM: *P*=.001). Between-group differences were significant for AM direction (*P*=.042) but not significant for MED (*P*=.173) or PM (*P*=.165) directions. Group-by-time interaction showed significant improvements for the 3 reach directions (*P*<.005).	1. The researcher administering the pre- posttest measurements was not blinded to participants’ group allocation. 2. Units of measurement were not reported; parameters of calculation were not reported.
Melam et al^46^	30	M:30	Exp: 21.0±2.2 Control: 21.3±2.3	1) Elastic tubing (front pull, back pull, crossover, reverse cross over) and 2) Physiotherapy conventional exercise program: - Range of motion and stretching exercises - Balance board activities - Heel and toe walking - Tandem walking.	Unaffected limb Affected limb was weight-bearing foot during exercises.	4 times/wk for 4 wk. 4 sets of 20 reps.	Regular conventional physiotherapy program.	SEBT	NA	Within-group changes in experimental group showed a significant improvement for the 3 reach directions (AM, MED, PM) (*P*<.05). Between-group differences were significant for all distances (*P*<.05). ESs suggest large practical significance, and the 95% CIs did not cross 0 for any direction. AM: ES=0.7; 95% CI, −2.0 to −1.7 MED: ES=0.6; 95% CI, −2.80 to −1.4 PM: ES=0.6; 95% CI, −2.5 to −1.4.	1. All male participants. 2. No follow-up performed.
Sierra-Guzmán et al^47^	50	M:33 F:17	VIB: 22.4±2.6 NVIB: 21.8±2.1 Control: 23.6±3.4	Repetition-based progression balance training program. VIB: unilateral balance training on BOSU ball on a vibration platform. NVIB group: unilateral balance training on BOSU ball on the floor.	Affected limb In participants with bilateral ankle instability, the ankle with the lower score on the CAIT was selected.	3 times/wk for 6 wk. 3 sets of four 45-s bouts of each exercise with 45-s rest between exercises. Frequency was increased by 5 Hz every 2 wk. Amplitude was increased from 2 to 4 mm after the first wk and then remained for the rest of the study.	Normal PA & no new PA.	SEBT	6 wk	Moderate to large ESs were present in several directions immediately postintervention between the VIB and Control groups (MED: ES=0.61; PM: ES=0.73; Composite: ES=0.54) and NVIB and Control groups (AM: ES=0.82; MED: ES=0.58; PM: ES=0.75; Composite: ES=0.80). Within-group analysis of VIB group showed moderate to large ESs between pre- and immediately postintervention: MED: ES=0.85; 95% CI, 0.97 to 7.69 PL: ES=0.52; 95% CI, 0.07 to 7.88 Composite: ES=0.68; 95% CI, 0.60 to 5.63 and Decreases between immediately after intervention and 6-wk follow-up: MED: ES=−0.43; 95% CI, −5.30 to −0.27 PL: ES=−0.38; 95% CI, −5.75 to −0.14 Composite: ES=−0.47; 95% CI, −3.68 to −0.48. In the NVIB group, moderate to large ESs were shown between pre- and immediately postintervention: MED: ES=0.78; 95% CI, 2.34 to 9.27 PM: ES=0.83; 95% CI, 2.38 to 11.79 PL: ES=0.43; 95% CI, 0.32 to 8.37 Composite: ES=0.58; 95% CI, 2.04 to 7.22. Decreases between immediately postintervention and follow-up were noted: A: ES=−0.40; 95% CI, −6.25 to −0.70 AM: ES=−0.39; 95% CI, −4.76 to −0.96 MED: ES=−0.47; 95% CI, −6.81 to −1.64 PM: ES=−0.40; 95% CI, −6.60 to −1.27 PL: ES=−0.35; 95% CI, −6.37 to −0.60 and Composite: ES=−0.41; 95% CI, −5.24 to −1.95.	1. Small sample size, might not have been adequate to detect postintervention differences among groups. 2. Participants were not blinded to group allocation.
Smith et al^39^	26	M:12 F:14	20.9±1.5	Hip strength protocol using progressive resistance exercises with TheraBand for hip internal rotation and abduction.	Affected limb	3 times/wk for 4 wk. 3 sets of 20 reps.	No intervention. Participants were not allowed engage in new lower extremity rehabilitation for the duration of the study.	Y Balance Test	NA	Between-group differences were significant for A (*P*<.01), PM (*P*<.01), and PL (*P*<.01) directions. Within-group changes in experimental group showed a significant improvement for the 3 reach directions (*P*<.001). Within-group displayed moderate to large ES, and 95% CI did not cross 0 for any direction for intervention group. A: ES=0.8; 95% CI, 0.0 to 1.6 PM: ES=1.1; 95% CI, 0.3 to 2.3 PL: ES=0.9; 95% CI, 0.1 to 1.7.	1. The assessing clinician was not blinded to the group allocation of the participants.

Abbreviations: A, anterior; AL, anterolateral; AM, anteromedial; BTP, balance training protocol; CAI, chronic ankle instability; CAIT, Cumberland Ankle Instability Tool; CI, confidence interval; DF, dorsiflexion; DLS, double-limb stance; D1, diagonal 1 movement pattern; D2, diagonal 2 movement pattern; ES, effect size; EVN, eversion; Exp, experimental group; F, female; FAAM, Foot and Ankle Ability Measure; FAAM-S, Foot and Ankle Ability Measure–Sport; INV, inversion; L, lateral; M, male; MED, medial; NA, not applicable; NVIB, nonvibration; P, posterior; PA, physical activity; PF, plantarflexion; PHSB, progressive hop-to-stabilization balance; PL, posterolateral; PM, posteromedial; PNF, proprioceptive neuromuscular facilitation strength protocol group; RBP, resistance band protocol; SL, single-limb; SLB, single-limb balance; SLS, single-limb stance; STARS, sensory-targeted ankle rehabilitation strategies; STP, strength training protocol; VIB, vibration.

### Description of studies

All studies provided demographic details and included 177 male and 191 female participants (mean weighted age, 23y) (see table 4). Four studies involved unilateral chronic ankle instability,^39^^,^^40^^,^^45^^,^^46^ and 6 investigated unilateral and bilateral chronic ankle instability.^35^, ^36^, ^37^, ^38^^,^^44^^,^^47^ Comprehensive baseline and follow-up data were presented in most cases (table 5).

**Table 5:** Data extracted from studies and calculated percentage change

Article Info	Intervention Group Baseline Scores,mean ± SD, measurements are normalized to leg length (%)									Calculated Percentage Change (%)											CorrespondingAverage MDC
SEBT Reach Directions										SEBT Reach Directions											Value (Table 4) (%)
Author(s)	A	AL	L	PL	P	PM	MED	AM	Composite	A	AL	L		PL		P	PM	M	AM	Average	
Anguish and Sandrey^35^	PHSB: 87.43±4.39 SLB: 83.93±5.71			PHSB: 88.96±3.50 SLB: 89.31±5.21		PHSB: 97.98±4.36 SLB: 95.8±6.71				PHSB5.71 SLB: 4.77				PHSB: 4.63 SLB: 5.58			PHSB: 3.23 SLB: 4.31			PHBS: 4.52 SLB: 4.89	4.48
Burcal et al^36^	BT: 63.36±9.34 BTS: 63.07±6.00			BT: 75.97±11.88 BTS: 71.60±10.35		BT: 79.67±8.91 BTS: 77.43±7.73				BT: 8.76 BTS: 4.91				BT: 9.02 BTS: 12.11			BT: 8.94 BTS: 9.67			BT: 8.9 BTS: 8.89	4.48
Cloak et al^45^	75.5±7.1	68.5±9.4	78.9±11.6	85.4±10.8	87.6±10	88.9±9.3	84.8±8	81±5.5		6.23	15.91	15.46		9.84		7.19	9.11	8.49	4.94	9.64	6.4
Cruz-Diaz et al^40^	76.47±5.13			78.99±1.51		82.35±2.55				4.79				5.67			5.26			5.24	4.48
Hall et al^38^									RBP: 97.4±7.2 PNF: 96.9±7											RBP: 4.72 PNF: 4.75	7.7
Hall et al^37^	Raw data not presented									Raw data not presented											
Linens et al^44^						0.85±0.12*	0.83±0.10*	0.83±0.08*									15.2	16.8	10.8	14.3	5.63
Melam et al^46^						91.8±4.2	89.5±5.0	84.3±3.2									2.18	2.35	2.49	2.34	5.63
Sierra-Guzmán et al^47^	VIB: 81.96±6.68 NVIB: 83.42±7.32			VIB: 87.34±9.64 NVIB: 88.73±10.65		VIB: 94.52±9.16 NVIB: 91.81±10.46	VIB: 89.74±5.49 NVIB: 88.09±9.95	VIB: 85.02±5.39 NVIB: 86.70±7.48	VIB: 87.72±5.63 NVIB: 87.75±7.24	VIB: 1.34 NVIB: 2.96				VIB: 4.56 NVIB: 4.89			VIB: 4.50 NVIB: 7.71	VIB: 4.83 NVIB: 6.60	VIB: 2.27 NVIB: 3.99	VIB: 3.55 NVIB: 5.28	5.61
Smith et al^39^	85.7±8.6			83.0±14.1		83.9±10.9				8.63				14.94			14.78			12.8	4.48


Article Info	Intervention Group Postintervention Scores, mean ± SD, measurements are normalized to leg length (%)																				
SEBT Reach Directions																					
Author(s)	A	AL	L	PL	P	PM	M	AM	Composite												
Anguish et al^35^	PHSB: 92.42±4.50 SLB: 87.93±5.17			PHSB: 93.08±3.80 SLB: 94.38±6.21		PHSB: 101.14±4.33 SLB: 99.93±6.18															
Burcal et al^36^	BT: 68.91±7.94 BTS: 66.16±6.29			BT: 82.82±11.02 BTS: 80.7±6.97		BT: 86.79±9.12 BTS: 84.92±8.08															
Cloak et al^45^	80.2±7.2	79.4±8.5	91.1±12.3	93.8±11.6	93.9±14.2	97±13.5	92±12.5	85±9.2													
Cruz-Diaz et al^40^	80.13±5.59			83.47±2.44		86.68±3.15															
Hall et al^38^									RBP: 102±7.2 PNF: 101.5±7.2												
Hall et al^37^	Raw data not presented																				
Linens et al^44^						0.98±0.11*	0.97±0.10*	0.92±0.11*													
Melam et al^46^						93.8±4.0	91.6±4.6	86.4±3.2													
Sierra-Guzmán et al^47^	VIB: 83.06±5.36 NVIB: 85.89±9.71			VIB: 91.32±7.63 NVIB: 93.07±10.07		VIB: 98.77±6.37 NVIB: 98.89±8.57	VIB: 94.07±5.07 NVIB: 88.09±9.95	VIB: 8.95±4.94 NVIB: 90.16±7.76	VIB: 90.83±4.33 NVIB: 92.38±7.27												
Smith et al^39^	93.1±7.4			95.4±11.1		96.3±8.9															

Abbreviations: A, anterior; AL, anterolateral; AM, anteromedial; BT, balance training; BTS, balance training with sensory-targeted ankle rehabilitation strategies; L, lateral; MED, medial; NVIB, nonvibration; P, posterior; PL, posterolateral; PHSB, progressive hop-to-stabilization balance; PM, posteromedial; PNF, proprioceptive neuromuscular facilitation strength protocol group; RBP, resistance band protocol; SLB, single-limb balance; VIB, vibration.

⁎ Units of measurement not reported.

Control groups included normal activity,^38^^,^^45^^,^^47^ general activity with strength training,^40^ bicycle workout,^37^ conventional physiotherapy,^46^ no intervention,^39^^,^^44^ or an active comparator.^35^^,^^36^ Two studies included a follow-up period, both short-term.^36^^,^^47^

### Risk of bias

**Fig 2:**
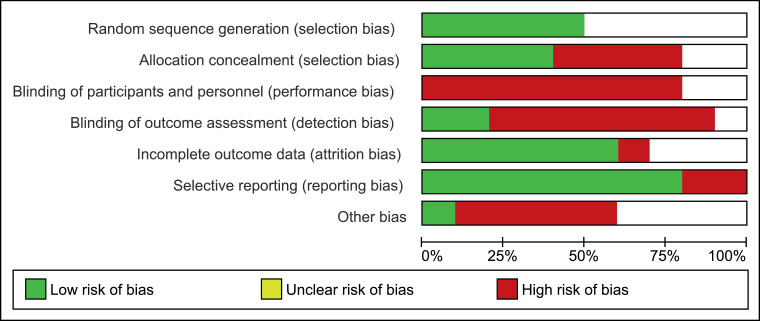
Risk of bias across studies.

Tables 6 and 7 present the outcomes of the Cochrane Risk of Bias Tool and the van Tulder scale. The mean score of the van Tulder scale was 5.1 of 11. Five studies had a high risk of bias,^37^^,^^38^^,^^44^, ^45^, ^46^ potentially caused by inadequate participant blinding, which is difficult to achieve with exercise interventions. This should be considered when interpreting these results. Common methodological shortcomings were inadequate allocation concealment^37^, ^38^, ^39^^,^^44^, ^45^, ^46^ and inadequate blinding of assessors and participants^35^, ^36^, ^37^, ^38^, ^39^^,^^44^, ^45^, ^46^ (fig 2). Only 4 studies reported both randomization and allocation concealment.^35^^,^^36^^,^^40^^,^^47^ Further shortfalls were lack of intention-to-treat analysis^37^, ^38^, ^39^^,^^44^^,^^46^^,^^47^ and failure to measure compliance.^36^^,^^39^^,^^45^, ^46^, ^47^

**Table 6:** Outcomes of Cochrane Risk of Bias Tool

Author(s)	Random Sequence Generation	Allocation Concealment	Blinding (Participants and Personnel)	Blinding (Outcome Assessor)	Addressed Incomplete Outcome Data	Free of Selective Reporting	Free of Other Sources of Bias	High/Moderate/Low Risk
Anguish and Sandrey^35^	Y	Y	N	N	Y	Y	U	Low
Burcal et al^36^	Y	Y	N	N	N	Y	N	Moderate
Cloak et al^45^	U	N	N	N	U	Y	N	High
Cruz-Diaz et al^40^	Y	Y	N	Y	Y	Y	Y	Low
Hall et al^38^	U	N	U	N	Y	N	U	High
Hall et al^37^	U	N	N	N	Y	N	N	High
Linens et al^44^	U	U	U	N	U	Y	U	High
Melam et al^46^	U	U	N	U	U	Y	N	High
Sierra-Guzmán et al^47^	Y	Y	N	Y	Y	Y	U	Low
Smith et al^39^	Y	N	N	N	Y	Y	N	Moderate

Abbreviations: N, no; U, unclear; Y, yes.

**Table 7:** Outcomes of van Tulder scale

Author(s)	Randomization	Allocation Concealment	Similar Baseline Characteristics	Patient Blinding	Investigator Blinding	Outcome Assessor Blinding	Cointervention Avoided	Compliance Acceptable		Dropout Rate Addressed	Intention-to-Treat Analysis	End Point (Similar Outcome Timing)	Total
Anguish and Sandrey^35^	Y	Y	U	N	Y	N	N		Y	Y	Y	Y	7
Burcal et al^36^	Y	Y	Y	N	N	N	Y		U	U	U	Y	5
Cloak et al^45^	U	N	Y	N	N	N	Y		U	U	U	Y	3
Cruz-Diaz et al^40^	Y	Y	Y	N	N	Y	N		Y	Y	U	Y	7
Hall et al^38^	U	N	Y	U	N	N	Y		Y	Y	N	Y	5
Hall et al^37^	U	N	U	N	N	N	Y		Y	Y	N	Y	4
Linens et al^44^	U	U	Y	U	U	N	Y		Y	U	N	Y	4
Melam et al^46^	U	U	Y	U	U	U	Y		U	U	N	Y	3
Sierra-Guzmán et al^47^	Y	Y	Y	N	Y	Y	Y		U	Y	N	Y	8
Smith et al^39^	Y	N	Y	N	N	N	Y		U	Y	N	Y	5

Abbreviations: N, no; U, unclear; Y, yes.

### Quality assessment

The 10 studies included 2 high-quality,^35^^,^^40^ 1 moderate-quality,^47^ and 7 low-quality studies^36^, ^37^, ^38^, ^39^^,^^44^, ^45^, ^46^ (table 8).

**Table 8:** Quality assessment results

Author(S)	Adequate Randomization	Adequate Allocation Concealment	Blinding of Assessors	Intent-to-Treat Analysis	Measurement of Compliance	Van Tulder Criteria Score	High/Moderate/LowQuality
Anguish and Sandrey^35^	Y	Y	N	Y	Y	7	High
Burcal et al^36^	Y	Y	N	U	U	5	Low
Cloak et al^45^	U	N	N	U	U	3	Low
Cruz-Diaz et al^40^	Y	Y	Y	U	Y	7	High
Hall et al^38^	U	N	N	N	Y	5	Low
Hall et al^37^	U	N	N	N	Y	4	Low
Linens et al^44^	U	U	N	N	Y	4	Low
Melam et al^46^	U	U	U	N	U	3	Low
Sierra-Guzmán et al^47^	Y	Y	Y	N	U	8	Moderate
Smith et al^39^	Y	N	N	N	U	5	Low

Abbreviations: N, no; U, unclear; Y, yes.

### Description and effectiveness of interventions

Four types of rehabilitation were investigated: balance,^35^, ^36^, ^37^^,^^44^ strength,^38^^,^^39^ vibration,^45^ and mixed training.^37^^,^^40^^,^^46^^,^^47^ Table 5 presents the data extracted from the included studies.

Balance programs included multidirectional hopping,^35^, ^36^, ^37^ progressive single-limb activities,^35^, ^36^, ^37^ wobble board exercises,^44^ and single-limb stance on different surfaces.^40^

Three studies implemented balance programs, 1 high quality^35^ and 2 low quality because of lack of blinding of assessors^36^ and inadequate allocation concealment.^44^ Anguish and Sandrey^35^ reported significant improvements in the SEBT for the progressive hop-to-stabilization and single-limb balance programs yielding an average percentage change of 4.52% and 4.89%, respectively, exceeding the corresponding MDC value of 4.48% (see table 5), indicating that these programs are effective.

Burcal et al^36^ reported significant improvements in the SEBT producing an average percentage change of 8.9%, again exceeding the corresponding MDC value of 4.48 %. These authors reported that balance training alone showed an equal magnitude of change as balance training with sensory-targeted ankle rehabilitation strategies (average percentage change: 8.89%).

Linens et al^44^ reported significant improvements in the SEBT for the wobble board group, producing the largest average percentage change of 14.3%, exceeding the corresponding MDC value of 5.63%, indicating that this program is effective. However, this is a low-quality study because of lack of allocation concealment; therefore, these results should be interpreted with caution.

Strength training included resistance band exercises for the ankle,^37^^,^^38^ resistance TheraBand exercises for the hip,^39^ proprioceptive neuromuscular facilitation training,^37^ and single-leg heel raises.^37^

Two studies conducted a strength program; both were deemed low-quality because of inadequate allocation concealment and lack of blinding of assessors.^38^^,^^39^ Hall et al^38^ reported that the resistance band group showed no significant improvements in the Y Balance Test, yielding a percentage change of 4.72%, which does not exceed their MDC of 7.7%, suggesting that this program is of limited effectiveness.

Smith et al^39^ reported significant improvements in the SEBT for their hip strengthening group yielding an average percentage change of 12.8%, substantially exceeding the corresponding MDC value of 4.48%, suggesting that this program is effective

One study implemented a vibration program involving single-leg heel raises and single-leg squats on a vibration platform.^45^ Cloak et al^45^ reported significant differences in the SEBT for the WBVT group compared with the controls, producing an average percentage change of 9.64%, exceeding the corresponding MDC value of 6.4%, again suggesting that the program is effective. This study is low quality because of inadequate allocation concealment; therefore, results should be interpreted with caution.

Three studies adopted a mixed training intervention incorporating general strength and balance training,^40^ elastic tubing exercises and conventional physiotherapy,^46^ and balance and vibration training.^47^

One study was deemed high quality,^40^ 1 moderate- quality,^47^ and 1 low quality.^46^ Cruz-Diaz et al^40^ reported significant improvements in the SEBT for the combined training group producing an average percentage change of 5.24%, exceeding the corresponding MDC value, suggesting that this program is effective.

Melam et al^46^ reported significant improvements in the SEBT for the mixed training group producing an average percentage change of 2.34%, which does not surpass the corresponding MDC value of 5.63%. Sierra-Guzmán et al^47^ reported significant improvements in the combined training group and the balance only training group, producing an average percentage change of 3.5% and 5.28%, respectively, which does not meet the corresponding MDC value of 5.63%, indicating that these programs are not effective.

One study compared balance and strength training^37^ and reported large effect sizes for both groups, with the balance group displaying slightly greater effects than the strength training group. However, this study was low quality because of inadequate allocation concealment; therefore, results should be interpreted cautiously.

### Intervention duration

Five studies involved a 4-week intervention,^35^^,^^36^^,^^39^^,^^44^^,^^46^ and 5 studies implemented a 6-week intervention.^37^^,^^38^^,^^40^^,^^45^^,^^47^ The average percentage change of the studies that included a successful 4-week^35^^,^^36^^,^^39^^,^^44^ and 6-week intervention^37^^,^^40^^,^^45^ was 13.4% and 7.44%, respectively. Hall et al^37^ did not provide data to calculate the average percentage and therefore is not comparable. These results suggest that 4 weeks of rehabilitation intervention is a sufficient duration to produce results that are clinically significant.

### Session frequency

One study completed 1 session per week,^45^ 8 completed 3 sessions per week,^35^, ^36^, ^37^, ^38^, ^39^, ^40^^,^^44^^,^^47^ and 1 completed 4 sessions per week.^46^

Successful interventions included a frequency of 1 session^45^ and 3 sessions per week,^35^, ^36^, ^37^^,^^39^^,^^40^^,^^44^ with an average percentage change of 9.64% and 11.49%, respectively. Hall et al^37^ did not provide data to calculate the average percentage and therefore is not comparable. These results suggest th\at 3 weekly sessions are sufficient to produce results that are clinically meaningful.

### Long-term effects

Burcal et al^36^ reported that improvements were maintained at the 1-week follow-up displaying effect sizes of 0.49 (anterior), 0.92 (posteromedial), and 1.42 (posterolateral). Whereas Sierra-Guzmán et al^47^ reported decreases from postintervention to the 6-week follow-up displaying composite effect sizes of −0.47 (vibration group) and −0.41 (nonvibration group).

## Discussion

This review aimed to determine (1) the effectiveness of rehabilitation for chronic ankle instability as measured by the SEBT and (2) the relative efficacy and the long-term effects of these rehabilitation interventions. The results suggest that rehabilitation of chronic ankle instability that includes wobble board exercises (average percentage change: 14.3%)^44^ and hip strengthening exercises (average percentage change: 12.8%)^39^ is the most effective because of a larger magnitude of change reported.

The benefits of wobble board rehabilitation for chronic ankle instability have been well-documented.^68^, ^69^, ^70^, ^71^ Strom et al^72^ investigated peroneal muscle activity and frontal plane ankle kinematics during a single-leg stance on different surfaces. They reported that the wobble board produced the largest improvements in neuromuscular abilities and ankle sensorimotor control.^72^ Research emphasizes that rehabilitation programs for chronic ankle instability should consider including wobble board exercises.^68^, ^69^, ^70^, ^71^^,^^73^^,^^74^

Previous research has reported that those with chronic ankle instability rely more on the hip's contribution during postural control tasks.^75^^,^^76^ Individuals with chronic ankle instability display insufficiencies in hip external rotators and gluteus medius function.^77^, ^78^, ^79^, ^80^ Therefore, highlighting that rehabilitation programs should consider including hip strengthening exercises.

The suggested optimal frequency is 3 sessions per week (average percentage change: 11.49%). This is supported by 2 high-quality^35^^,^^40^ and 4 low-quality studies.^36^^,^^37^^,^^39^^,^^44^ The suggested optimal duration is 4 weeks (average percentage change: 13.4%). This result is supported by only 1 high-quality^35^ and 3 low-quality studies.^36^^,^^39^^,^^44^ Similarly, Powden et al^81^ reported that improvements observed in individuals with chronic ankle instability after a 4-week multimodal intervention were equal to that of a 6-week intervention.

### Relative efficacy of rehabilitation types

Only 1 study compared different rehabilitation types^37^ and reported that balance training was slightly more effective. However, because of this study being low quality and the lack of studies that compared different rehabilitation types, these results are not conclusive.

### Long-term effects

Burcal et al^36^ reported that improvements were maintained at the 1-week follow-up displaying moderate to large effect sizes. Whereas Sierra-Guzmán et al^47^ reported decreases at the 6-week follow-up with the nonvibration group displaying better ability to maintain postintervention improvements. Because of the lack of long-term follow-up assessments, the long-term effects of the interventions are unknown.

### Comparison to previous literature

This is the only review in the last 10 years that has assessed the effectiveness of different rehabilitation types and suggested optimal rehabilitation parameters. Before this review, Webster and Gribble^60^ investigated functional rehabilitation interventions for chronic ankle instability published from 1988-2008. They analyzed postural control outcome measurements in 6 studies,^60^ reporting that a 4- to 6-week intervention with 3-5 weekly sessions can improve dynamic postural control. Webster and Gribble,^60^ similar to this review, found wobble board rehabilitation effective for several stages of ankle instability.^60^ Unlike this review, Webster and Gribble^60^ assessed methodological quality of their studies using the Physiotherapy Evidence Database scale. However, the Physiotherapy Evidence Database scale has many short comings; it assesses the quality of reporting instead of characteristics that affect the risk of bias (which is recommended^63^^,^^82^) and does not account for compliance or timing of outcomes, which are important when evaluating exercise interventions.

### Study limitations

The importance of postural control is accepted for many clinical populations^83^; however, the population of interest in this review was recreational athletes, and therefore the results may not be applicable to more general clinical groups. Second, the accumulated number of participants assessed is relatively small; including a study with a small sample size could have significantly influenced the magnitude of change between pre- and postintervention scores. Third, this review only analyzed dynamic postural control; by incorporating self-reported measures this may have provided a more in-depth functional rehabilitation program for chronic ankle instability. Lastly, because a meta-analysis was not conducted, the findings of this review can only suggest optimal rehabilitation parameters; they are not conclusive.

Despite these limitations, this review rigorously evaluated risk of bias within and across the included studies. Furthermore, this is the only review that discusses an optimal rehabilitation program for recreational athletes with chronic ankle instability, thus assisting clinicians regarding the conservative management of chronic ankle instability.

### Recommendations for future research

Future trials should be adequately powered and focus on meeting the minimum standards to reduce potential threats to bias. There is a need for trials to directly compare different rehabilitation types to provide a definite conclusion regarding the relative efficacy. Future trials should include a sufficient follow-up period to determine the long-term effects of an intervention.

### Clinical relevance

This review suggests the optimal rehabilitation parameters required in the management of recreational athletes with chronic ankle instability. Three weekly sessions focusing on wobble board exercises and hip strengthening for 4-6 weeks is suggested. However, the evidence is insufficient for these results to be conclusive and are only suggestions to help guide clinicians in the management of chronic ankle instability.

## Conclusions

Chronic ankle instability is associated with impaired sensorimotor control, which contributes to deficits in postural control activities. A rehabilitation approach focusing on wobble board exercises and hip strengthening performed 3 times weekly for 4-6 weeks is suggested to help improve dynamic postural control in recreational athletes with chronic ankle instability, at least in the short-term. The lack of long-term follow-up studies prevents definitive conclusions, and the results are suggested as a guideline to assist clinicians in the management of recreational athletes with chronic ankle instability. The long-term effects of the interventions remain unclear and further research is required.
